# Framing Cause-Effect Relationship of Acute Coronary Syndrome in Patients with Chronic Kidney Disease

**DOI:** 10.3390/diagnostics11081518

**Published:** 2021-08-23

**Authors:** Mădălina Ioana Moisi, Simona Gabriela Bungau, Cosmin Mihai Vesa, Camelia Cristina Diaconu, Tapan Behl, Manuela Stoicescu, Mirela Mărioara Toma, Cristiana Bustea, Cristian Sava, Mircea Ioachim Popescu

**Affiliations:** 1 Department of Preclinical Disciplines, Faculty of Medicine and Pharmacy, University of Oradea, 410073 Oradea, Romania; mada_vidican@yahoo.ro (M.I.M.); cristianabustea@yahoo.com (C.B.); 2 Department of Pharmacy, Faculty of Medicine and Pharmacy, University of Oradea, 410028 Oradea, Romania; mire.toma@yahoo.com; 3 Doctoral School of Biological and Biomedical Sciences, University of Oradea, 410087 Oradea, Romania; 4 Department 5, “Carol Davila” University of Medicine and Pharmacy, 050474 Bucharest, Romania; drcameliadiaconu@gmail.com; 5 Department of Internal Medicine, Clinical Emergency Hospital of Bucharest, 105402 Bucharest, Romania; 6 Department of Pharmacology, Chitkara College of Pharmacy, Chitkara University, Punjab 140401, India; tapanbehl31@gmail.com; 7 Department of Medical Disciplines, Faculty of Medicine and Pharmacy, University of Oradea, 410073 Oradea, Romania; manuela_stoicescu@yahoo.com (M.S.); cristian.sava2004@gmail.com (C.S.); procardia_oradea@yahoo.com (M.I.P.)

**Keywords:** chronic kidney disease, non-traditional risk factors, ischemic coronary artery disease, accelerated atherosclerosis, acute coronary syndromes

## Abstract

The main causes of death in patients with chronic kidney disease (CKD) are of cardiovascular nature. The interaction between traditional cardiovascular risk factors (CVRF) and non-traditional risk factors (RF) triggers various complex pathophysiological mechanisms that will lead to accelerated atherosclerosis in the context of decreased renal function. In terms of mortality, CKD should be considered equivalent to ischemic coronary artery disease (CAD) and properly monitored. Vascular calcification, endothelial dysfunction, oxidative stress, anemia, and inflammatory syndrome represents the main uremic RF triggered by accumulation of the uremic toxins in CKD subjects. Proteinuria that appears due to kidney function decline may initiate an inflammatory status and alteration of the coagulation—fibrinolysis systems, favorizing acute coronary syndromes (ACS) occurrence. All these factors represent potential targets for future therapy that may improve CKD patient’s survival and prevention of CV events. Once installed, the CAD in CKD population is associated with negative outcome and increased mortality rate, that is the reason why discovering the complex pathophysiological connections between the two conditions and a proper control of the uremic RF are crucial and may represent the solutions for influencing the prognostic. Exclusion of CKD subjects from the important trials dealing with ACS and improper use of the therapeutical options because of the declined kidney functioned are issues that need to be surpassed. New ongoing trials with CKD subjects and platelets reactivity studies offers new perspectives for a better clinical approach and the expected results will clarify many aspects.

## 1. Introduction

Stable ischemic coronary artery disease (CAD) and ACS have a high incidence in the general population. The mortality rate induced by coronary heart disease is significant, imposing a constant update and improvement of diagnostic and treatment algorithms [1].

Chronic kidney disease (CKD), in all stages of evolution, is associated with a significant cardiovascular risk (CVR), and patients diagnosed with this condition are more likely to develop ACS or malignant rhythm disorders with a fatal prognosis. Thus, the deaths of patients with CKD are predominantly caused by cardiovascular (CV) comorbidities and less by the progression of kidney disease. Patients with end-stage CKD have a CV death rate of approximately 50% [2].

According to the American Heart Association Councils on Kidney in Cardiovascular Disease, High Blood Pressure Research, Clinical Cardiology, Epidemiology and Prevention, the incidence of ACS was of 40% in subjects who required initiation of renal replacement therapy and changes such as concentric left ventricular hypertrophy (CLVH) or echocardiographic abnormalities were detected in 85% of patients [3].

Atherosclerosis is a complex process, initiated by risk factors (RF) such as dyslipidemia, smoking, family history of premature CAD, personal history of diabetes mellitus (DM) or hypertension (HTN), and overweight. The above RF also initiate an inflammatory reaction, responsible for the activation of monocytes and macrophages [4].

In case of a discrepancy between the myocardial oxygen demand and supply, ischemia occurs, more prominent at subendocardial level. The perfusion of the subendocardial area is performed in diastole and ischemia accentuates the decrease of the coronary perfusion pressure, significantly influencing the subendocardial cells, whose vascularization is dependent on the value of this pressure. The perfusion of subepicardial cells is performed in both systole and diastole, and the decrease in coronary perfusion pressure causes ischemia only in the case of a drastic decrease of this pressure to values below 25 mmHg [4].

This review aims to present the current knowledge of accelerated ACS in patients with CKD. Their unfavorable prognosis can be improved by identifying and influencing the mechanisms responsible for atherosclerosis, with different characteristics in the uremic environment context. The exposure of all pathophysiological links involved in the etiology of CAD in patients with CKD, including novelties in this area, is an asset of this paper. Moreover, the unfavorable evolution of patients with end-stage renal disease (ESRD) and ACS, along with the lack of studies to guide the proper management of these diseases, justify our choice regarding this topic, but also the need for all information centralized in this manuscript, as a solid data base for further studies in the field.

## 2. Materials and Methods

**Figure 1:**
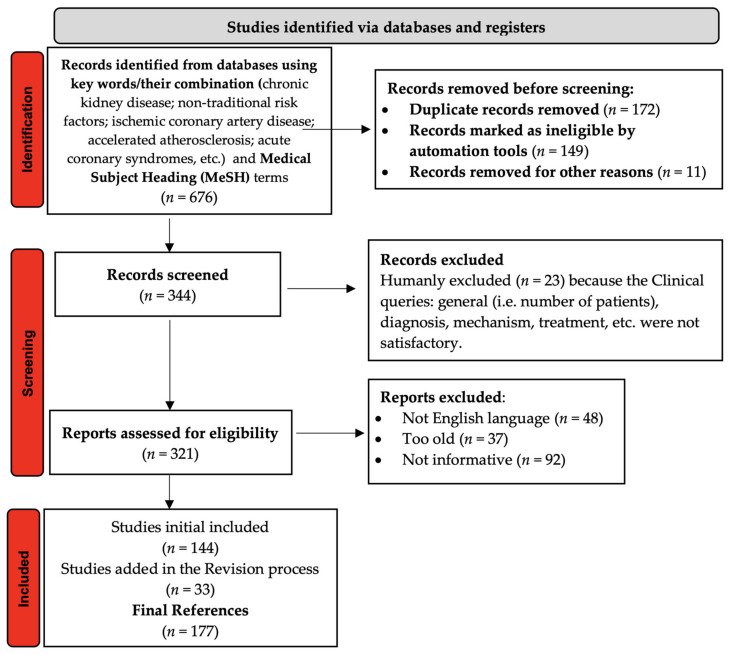
Flow chart on the selection process of bibliographic sources included in this article.

We performed an exhaustive research of the relevant literature and selected the scientific publications addressing atherosclerosis in patients with CKD, also emphasizing the most important pathophysiological aspects. There was no limitation on the publication dates of the selected articles, recent and old papers were equally analyzed, including articles published in 2021. The PubMed, Cochrane Library and Web of Science databases were searched for relevant information on the topic. The criteria used to select the necessary bibliography, as well as the description of the whole process is highlighted in Figure 1 (a PRISMA flow diagram), according to Page et al. recommendations [5,6]. The keywords mentioned at the beginning of this paper (“atherosclerosis in chronic kidney disease”, “acute coronary syndromes in chronic kidney disease”, “non-traditional risk factors”, etc.) and the Medical Subject Heading (MeSH) terms were used for finding the most appropriate studies. The articles considered eligible were initially selected based on both their title and abstract, then a complex analysis of their content (using filtering techniques like Clinical Queries) was performed, and the most relevant and informative data and results were extracted.

## 3. Development and Evolution of Cardiovascular Diseases—The Role of Independent Risk Factors Attributed to CKD

A study on 85 patients with CKD showed that the prevalence of CLVH is correlated with the progression of the decline in renal function, detecting signs of CLVH even before the introduction of renal replacement therapy [7].

The Framingham Heart Study was one of the first studies to highlight the association between impaired renal function and CV-induced mortality. Six thousand two hundred and twenty three participants without pre-existing CV conditions were included and followed for a period of 15 years, highlighting that male patients with mild CKD frequently had major adverse CV events (MACE) and an increased mortality rate [8].

The Ongoing Telmisartan Alone and in Combination with Ramipril Global Endpoint Trial (ONTARGET) and Telmisartan Randomized Assessment Study in ACE Intolerant Subjects with Cardiovascular Disease (TRANSCEND) illustrated that a low glomerular filtration rate (GFR) was associated with an increased albumin/creatinine ratio (ACR), amplifying the risk of developing CV diseases [9,10].

In a systematic review, Tonelli et al. included 1.3 million patients with CKD stages 3 and 4, followed for a period of 48 months, and excluded patients in need of renal replacement therapy. Patients with low GFR had the highest mortality compared to those with DM or myocardial infarction (MI) sequelae, even after adjustments for age, sex [11], and other comorbidities. Patients with no history of MI developed ACS more frequently if they had DM associated with CKD. It was, thus, highlighted that moderate and advanced CKD is equivalent to CAD in terms of death risk [12].

Two meta-analyses attempted to certify the role of independently assigned CVRF to CKD. It was hypothesized that decreased GFR and albuminuria are predictors of CV mortality, these analyses being performed in contrast to the influence of HTN and DM. The first meta-analysis included 1,127,656 patients and demonstrated that decreased GFR and marked albuminuria are predictors of mortality in normotensive patients. The increased mortality rate observed in the group of patients with CKD and albuminuria was not influenced by the presence of HTN. The authors concluded that CKD can be considered a RF that influences the death rate even in normotensive subjects [13]. The second meta-analysis included 1,024,977 participants, revealing that CV-induced mortality is associated with decreased GFR and the presence of albuminuria, independent of the association of DM [14].

Most studies showed that CKD induces an increase in the aortic arterial stiffness, promoting compensatory CLVH and diastolic dysfunction in patients with advanced CKD. An experimental study including two specific types of rodents illustrated the peculiarities of aortic arterial stiffness in the early and moderate stages of chronic renal dysfunction. Thus, the two types of rodents, a group whose gene expression responsible for the coding of apolipoprotein E was repressed and a group of wild-type C57 species, were assigned based on CKD association. Moderate stage of CKD has been shown to correlate with significant vascular calcifications that induce aortic arterial stiffness. The explanation lies in the synthesis of adhesion molecules such as intercellular adhesion molecule-1 (ICAM-1) and vascular cell adhesion protein 1 (VCAM-1), responsible for aortic stiffness, and structural changes in the heart that can be attributed to CKD, as well. The involvement of endothelial changes by altering the ratio between collagen and elastin in the arterial wall, hypercholesterolemia induced by repression of the gene encoding apolipoprotein E, or vascular calcifications do not have evidence of aortic stiffness appearance. Arterial stiffness is an emblem of CKD and has an increased incidence in case of significant decline in renal function [15]. The increase in aortic arterial stiffness caused by the vascular calcification process corroborated with non-traditional RF is observed in advanced stages of CKD.

The studies mentioned above illustrates the evidence that CKD may be responsible for CV disease occurrence. Despite the presence of the RF, some potential explanations regarding the correlation of CKD with CV disorders consist in the major role of the damaged kidney in synthesis of inflammatory mediators, hormones and enzymes [16]. These products will be responsible for vascular remodeling with a certain negative effect on the heart. Another important element involved in the association of CKD with CV disease is represented by accumulation of uremic toxins which may affect the CV system. Some experimental and clinical studies revealed that p-cresyl sulfate is responsible for endothelial damage and indoxyl sulfate stimulates the oxidative stress by entering the cardiac cell using a specific receptor [17].

Asymmetric dimethylarginine (ADMA) is a major uremic toxin which promotes an accelerated atherosclerosis and has an important contribution in development of CV disorders [18]. The high levels of ADMA discovered in subjects with mild impairment of the kidney function suggests that there is another potential mechanism that explains the accumulation of the compound, beside the decreased value of GFR [19]. The final element that is incriminated in CV disease pathogenesis in patients with CKD is the presence of proteinuria. The hypothesis that proteinuria promotes an important inflammatory status, endothelial disfunction and alteration of the coagulation- fibrinolysis balance was already demonstrated [20]. In the early stages of CKD, there is evidence of insulin resistance, which is responsible for activating the sympathetic nervous system and reducing natriuresis. These phenomena can be incriminated in the appearance of CVRF and RF specific to the uremic environment [21,22].

## 4. CKD—Equivalent to Ischemic Coronary Heart Disease in Terms of Risk of Death

There have been numerous experimental and clinical studies that have confirmed the role of independent RF of CKD exerting a major influence on the mortality rate in patients with ACS [23,24]. Thus, Shlipak and colleagues studied 130,099 patients with acute myocardial infarction (AMI) and found that mild renal dysfunction, defined by serum creatinine values between 1.5 and 2.4 mg/dL, and moderate renal dysfunction, characterized by serum creatinine values between 2.5 and 3.9 mg/dL, were independent predictors of the risk of death [25].

Suwaidi et al. analyzed patients with AMI from four clinical trials and found that CKD was associated with increased mortality at 30 days and 6 months compared to patients with preserved renal function, regardless of whether they had an AMI with ST-segment elevation (STEMI) or acute AMI without ST-segment elevation (NSTEMI) [26]. Furthermore, a much more recent study added new data on STEMI patients [27].

A prospective study on 13,329 patients without history of CAD, followed for a period of 9 years, showed that anemia and elevated serum creatinine (≥1.2 mg/dL in women or ≥1.5 mg/dL in men) increase the risk of AMI 2.7 times compared to patients with normal renal function [28].

All these observations outline that CKD should be considered the CAD equivalent in terms of mortality risk. The above evidence also confirms that CKD is an independent predictor of mortality in case of ACS occurrence. It has been shown that each decrease in GFR by 10 mL/min/1.73m^2^ increases CV mortality by 5% [29].

**Figure 2:**
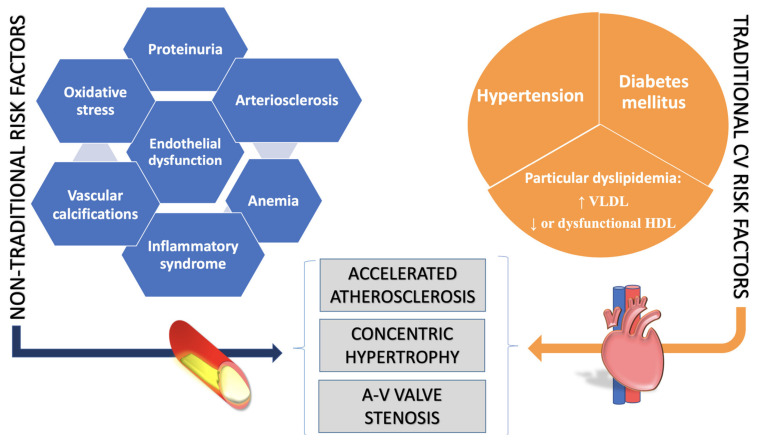
Consequences of the complex interaction between traditional CVRF and non-traditional RF specific to the uremic environment. VLDL—very low-density lipoproteins, HDL—high-density lipoproteins, A-V—atrioventricular.

The atherosclerotic process has special characteristics in patients with pre-existing CKD. Only the classic CVRFs do not explain the increased incidence of cardiac deaths in patients with CKD. Patients requiring renal replacement therapy show a U-shaped curve of the influence of blood pressure (BP) and lipid values on the occurrence of CAD and mortality, extreme variations having similar results. Thus, non-traditional RF were also highlighted: inflammation, anemia, proteinuria, endothelial dysfunction and alteration of phospho-calcium metabolism, incriminated in accelerated atherosclerosis of patients with CKD (Figure 2) [30].

The evolution of ACS in patients with CKD may be influenced by the appropriate dosage and interpretation of cardiac biomarkers in conjunction with a detailed echocardiographic examination, an aspect highlighted by a study that included both patients with STEMI and NSTEMI [31].

## 5. The Role of Dyslipidemia in Initiating the Process of Atherosclerosis in Patients with CKD

Regarding dyslipidemia, studies have shown that patients with CKD and marked proteinuria frequently have hypercholesterolemia. Decreased renal function is associated with increased triglyceride levels and decreased high-density lipoproteins (HDL). The lipid profile of patients with CKD reveals elevated values of very low-density lipoproteins (VLDL) and triglycerides. Abnormalities of the enzymes involved in lipid metabolism will determine an increase in the percentage of triglycerides of the mentioned particles [32].

ESRD associates elevated lipoprotein-a values, with significant atherogenic properties. CKD patients have dysfunctional HDL, triglyceride-rich fractions that amplify oxidative stress and inflammation [33].

Seliger and colleagues revealed that statin treatment of CKD-associated dyslipidemia decreased mortality rate by 32%, a percentage uninfluenced by other independent predictors such as DM, age, smoking status, or history of CV diseases [34]. Another study focused on statins in women revealed the newest data on this field [35].

The Assessment of Lescol in Renal Transplantation (ALERT) study included 2102 patients with kidney transplantation and elevated cholesterol, showing that fluvastatin administration reduced the number of cardiac deaths and risk of non-fatal AMI compared to the placebo group (70 vs. 104, *p* = 0.05). Although the primary objective of the study was not achieved and CV mortality was not substantially reduced, it was considered important to follow these patients for a period of 5 years to assess whether the statin treatment is not harmful in these subjects, and it may be a therapeutical option [36].

## 6. Proteinuria and Microalbuminuria—The Role Played in the Process of Atherosclerosis in CKD

Proteinuria and microalbuminuria are used in the definition of CKD [37], being predictors of increased CVR. The association between decreased GFR and proteinuria in patients with pre-existing CAD increases the probability of developing MACE and cardiac deaths by three times [38].

The Irbesartan Diabetic Nephropathy study included patients with HTN, DM and macroalbuminuria, defined as an ACR ratio >300 mg/g. The patients were divided into three subgroups, with distinct therapeutic approach: the first group received irbesartan, the second received amlodipine, and the third received placebo therapy. Irbesartan has been shown to be effective in slowing the progression to ESRD and maintaining a constant serum creatinine but did not achieve the desired effects on the prognosis and evolution of MACE [39].

A multivariate analysis highlighted the role of albuminuria as independent RF, being incriminated in the development of MACE. For each increase of the ACR ratio with a unit, the relative risk of MACE appearance increased by 1.3 times [40].

Another study, Reduction of Endpoints in NIDDM with the Angiotensin II Antagonist Losartan (RENAAL), had the same design as the study with irbesartan, but this time losartan was the chosen drug. This study failed to demonstrate a reduction in MACE, with differences only in the development of HF, which was lower in the losartan group [41]. A further analysis of the RENAAL study divided the patients into three groups, based on the ACR value, and showed that a reduction of albuminuria by 50% decreased the risk of MACE and CV death by 18% [42].

Mattock et al. have shown that microalbuminuria is the strongest predictor of CV diseases prognosis, being more sensitive than smoking status, diastolic blood pressure (DBP) or serum cholesterol. The analysis included patients with type 2 DM and aimed to identify the most accurate factor for predicting mortality caused by CAD [43].

The Heart Outcomes Prevention Evaluation (HOPE) trial found that microalbuminuria and ACR >2 mg/mmol, both in the DM population and in the subjects without DM, increased the risk of developing MACE by 1.83 times. The overall mortality also increased 2.09 times in patients with microalbuminuria [44].

The ability of microalbuminuria to shape CVR is not limited to patients already at high risk, such as the study population in the HOPE trial. There is evidence concerning the effectiveness of microalbuminuria in CVR stratification compared to other classical RF. A urinary microalbuminuria 2 times higher is associated with a relative risk of CV mortality of 1.29 and a risk of general cause death of 1.12 [45].

There is a hypothesis that CVR is present even at values defined as normal albuminuria (ACR <3 mg/mmol). The subjects of the HOPE study were also analyzed on this occasion, and the cut-off value of albuminuria considered benign in influencing CVR was 0.5 mg/mmol. For a minimal increase in albuminuria with 0.4 mg/mmol, there is an increase of the relative risk of MACE with 5.9% [46].

Klausen et al. also highlighted that the CVR in the general population also increases if albuminuria values are increased, but lower than the cut-off value for the diagnosis of microalbuminuria. The patients were divided into subgroups based on microalbuminuria values. It has been shown that urinary albumin excretion above 48 μg/min (equivalent to an ACR of 9 mg/g) is associated with a relative risk of developing CAD two times higher and a risk of CV death 1.9 times higher [47].

## 7. The Role of Inflammation in the Initiation of Atherosclerosis and Specific Features in Patients with CKD

Inflammation is another non-traditional RF, with a prevalence of about 50% in patients in the pre-dialysis stage and those who require dialysis. CKD patients have been shown to have elevated C-reactive protein (CRP) values. There are numerous studies that certify the independent CVRF status of the CRP biomarker in these patients [48]. With the destruction of functional nephrons, an inflammatory response is triggered, in the context of losing the role that the renal apparatus plays in maintaining homeostasis [49].

Inflammation may cause endothelial injury, endothelial dysfunction and atherosclerotic plaques formation [50]. Atherosclerosis can also induce an inflammatory response through oxidized low-density lipoprotein (LDL) particles that stimulate the synthesis of cytokines, interleukin 6 (IL-6), tumor necrosis factor α (TNF-α), interferon and nuclear factor κB [51,52]. Inflammation and oxidative stress are non-traditional RF, but they have been shown to represent the common pathway through which traditional CVRFs amplify the decline in renal function. Thus, uremic toxins will induce the production of free radicals in kidney cells, triggering an inflammatory response. In turn, the triggered inflammation can generate the synthesis of reactive oxygen species through macrophage cells [53]. There is a dynamic interaction between the inflammatory response and oxidative stress, the two processes being directly involved in the initiation of atherosclerosis and damage to kidney cells.

Highly sensitive C-reactive protein (hs-CRP) is a predictor used to estimate the risk of developing AMI, acute ischemic stroke (AIS) and CV death [54]. Even after adjustments for the influence of other CVRFs in low- and high-risk patients, respectively, the predictive power of this biomarker remains significant. The role of other inflammatory markers, such as IL-6, serum amyloid A, or intercellular adhesion molecules has been studied, but these have shown a lower predictive capacity than hs-CRP [55].

The study Justification for the Use of Statins in Prevention: an Intervention Trial Evaluating Rosuvastatin (JUPITER) included patients without CV history, but with hs-CRP values greater than 2 mg/L and illustrated the importance of statin therapy even in patients with normal lipids values, in order to reduce the inflammatory response. Thus, a 44% reduction in vascular events was demonstrated (*p* = 0.00001), and the AMI rate decreased by 54% (*p* = 0.0002). Also, the incidence of AIS decreased by 48% (*p* = 0.002) and the death rate from any cause decreased by 20% [56]. The conclusion of the study was that the administration of rosuvastatin in patients without a personal pathological history or dyslipidemia, but with elevated hs-CRP values, contributes to reducing the occurrence of MACE.

## 8. Oxidative Stress and Endothelial Dysfunction—Aspects Regarding the Pathogenesis of Atherosclerosis in the Context of CKD

Oxidative stress has a well-defined role in kidney cells injury. The increased synthesis of reactive oxygen species exceeds under certain conditions the inactivation capacity of antioxidants and thus will result in a prooxidant status. Oxidation of lipids, proteins and carbohydrates will trigger the progression of CKD [57]. The mechanism of triggering oxidative stress in CKD is the activation of the reduced form of nicotinamide adenine dinucleotide oxidase (NAD(P)H), xanthine-oxidase, myeloperoxidase, and nitric oxide (NO) synthetase inactivators. NAD(P)H is the most important prooxidant element in endothelial cells [58]. Myeloperoxidase is released by leukocytes during hemodialysis sessions, being incriminated in the vascular injury process highlighted in patients with ESRD [59].

A major factor in the initiation of atherosclerosis, endothelial dysfunction, decreases the availability of NO, the most potent vasodilator. NO synthesis is performed by an enzyme called NO synthetase [60]. Asymmetric dimethyl arginine (ADMA), an inhibitor of the enzyme responsible for NO synthesis, is an independent predictor of CV-linked mortality in CKD [61]. The serum level of ADMA is increased in patients with CKD, because the mechanism of inactivation and the excretion of the product are compromised. Coen has shown that serum ADMA levels may be influenced by the severity of hyperparathyroidism in CKD and are correlated with the CV death of these patients [62].

The study of Krzanowski et al. included 51 patients with CKD, in which an arteriovenous fistula has been created to initiate hemodialysis. A histological analysis of the collected renal arteries was performed, and the results were compared with the samples from 33 healthy volunteers. ADMA was an independent predictor in quantifying radial artery calcification. In the advanced stages of CKD, the serum level of ADMA was correlated with the presence of the middle arterial tunic calcification. Patients with arterial lesions in an advanced calcification stage had low ADMA values, suggesting that endothelial lesions caused by ADMA occurred early, and the vascular calcification process began later [63].

## 9. The Anemia Role in the Pathogenesis of Atherosclerosis in CKD

Anemia in patients with CKD is the result of a deficient synthesis of erythropoietin, generating an imbalance between the oxygen demand and supply in the cells [64]. CLVH in CKD is a consequence of hypervolemic status due to decreased renal water excretion. The presence of CLVH in conjunction with anemia considerably decreases the oxygen supply. Studies show that in patients with CKD, an increase in serum hemoglobin (Hb) from 7.9 to 10.4 g/dL improves exercise tolerance and is associated with a decrease in ST-segment elevation on electrocardiogram (ECG) [65].

The consequences of anemia are represented by decreased red blood cells counts (RBCs), plasma viscosity, and peripheral vascular resistance [66]. The venous return will increase, and hypoxemia at the cellular level will stimulate the sympathetic nervous system. The latter will act by increasing heart rate and cardiac output, with CLVH occurrence. If anemia persists and is not corrected, CLVH becomes maladaptive [67].

Clinical trials have attempted to demonstrate the benefit of correcting anemia in subjects with CKD and the effect in preventing the onset of CV disease. The first study enrolled 1200 patients with CAD or HF and anemia, characterized by low (30%) or normal (42%) and slightly low hematocrit (Hct) values, respectively. In patients with low Hct, an attempt was made to increase it by administering erythropoietin, and in the group of patients with normal Hct, erythropoietin was administered only to maintain the Hct value. The main objective of the study focused on the time elapsed until the diagnosis of AMI and CV death. Patients with low Hct had a favorable evolution after anemia correction [68].

The second study included 146 patients with CLVH, respectively eccentric left ventricular remodeling (LV), such as ventricular dilation and anemia. Patients benefited from the correction of anemia by erythropoietin administration and were divided into two groups, based on the target Hb value of 10 g/dL or 13 g/dL. Patients with CLVH have been shown to have the same LV mass index for both target values. The same conclusion was drawn for LV dilation, the difference being illustrated by the fact that subjects with CLVH were less likely to progress to LV dilation if they were in the group with higher Hb value [69].

Impaired renal function will reduce urinary phosphate excretion and hyper phosphatemia. The vascular calcification process highlighted in CKD subjects involves precipitation and deposition of phosphate and calcium complexes. Moreover, vascular smooth muscle cells will transform into osteoblast-like cells, activating the synthesis of the extracellular matrix. Increased phosphatemia by 1 mg/dL equates with a risk of CV mortality by CAD of approximately 9% [23].

The Second Manifestations of ARTerial Disease (SMART) study reveals that a decreased renal function and increased serum creatinine are often associated with non-obstructive atherosclerotic lesions [70].

## 10. Arteriosclerosis and Vascular Calcification—The Role in the Pathogenesis of ACS in Patients with CKD

Arteriosclerosis is the initial pathological process that appears in patients with CKD. This phenomenon illustrates a reduced arterial compliance caused by the decrease of the elastic tissue and activation of the vascular fibrosis process. The consequences of arteriosclerosis include diastolic dysfunction and CLVH. The increase in pulse wave velocity characteristic to patients with arterial stiffness is also highlighted in CKD. Changing aortic pulse wave velocity is an independent predictor of ESRD patients’ survival [71]. Arteriosclerosis interferes with the proper coronary perfusion that occurs in diastole, exacerbating the imbalance between oxygen demand and supply [72]. Another theory reflects the fact that despite myocyte hypertrophy, the volume of the capillaries remains constant, thus there is a discordance between the two elements [73].

Extensive vascular calcification is the specific element of atherosclerosis objectified in the case of a pre-existing CKD, the alteration of mineral metabolism being often incriminated [74]. Schwartz demonstrated the importance of vascular calcifications characteristic to CKD, highlighted in the middle tunic. The control group with preserved renal function showed distinct, fibrous atherosclerotic lesions. The evolution of these lesions is largely due to their composition, the size of the atheroma plaque not being significant [75]. Recent studies show a significant correlation between the extent of vascular calcifications, revealed by computed tomography, and the decrease of GFR below 60 mL/min/1.73 m^2^ [76].

The process of vascular calcification involves precipitation and deposition of calcium and phosphorus complexes in the middle arterial tunic, and the process characteristic to atherosclerosis targets the arterial intima and associates cell necrosis, inflammation, and lipid particle deposition. It has been shown that the process of vascular calcification at the level of the middle layer (tunica media) is found in diabetic patients and those diagnosed with CKD [77]. This process does not involve only the precipitation of phospho-calcium complexes and their passive deposition in the arteries, the essential element being the transformation of vascular smooth muscle cells into cells with osteoblast-like properties [78,79].

Reynolds concluded that hyperphosphatemia and the uremic medium are responsible for the apoptosis of smooth muscle cells in the arteries, generating apoptotic bodies capable of stimulating the process of vascular mineralization. The increased extracellular concentration of calcium amplifies their release and increases the expression of vesicles in the cell membranes, which initiates vascular calcification. Thus, even in the absence of the osteoblast-specific cellular conformation changing process, vascular smooth muscle cells undergo significant transformations in patients with ESRD [80].

Vascular calcifications and valvular diseases have an incidence of 40% in patients with CKD and GFR of 33 mL/min/1.73 m^2^, compared to 13% in those with preserved renal function [81].

Uremic toxins intervene in the process of vascular calcification by stimulating the secretion of a mediator essential for the process of transforming smooth muscle cells into osteoblasts, called bone morphogenetic protein 2 (BMP-2) [82].

Leptin is another promoter of vascular smooth muscle cell differentiation. The serum level of this hormone is increased because the renal excretion of this compound is compromised in patients with CKD. There are two mechanisms that explain the effect of leptin: excessive stimulation of the sympathetic nervous system, which activates beta-adrenergic receptors of osteoblasts, and an increased synthesis of the BMP-2 compound [83].

**Figure 3:**
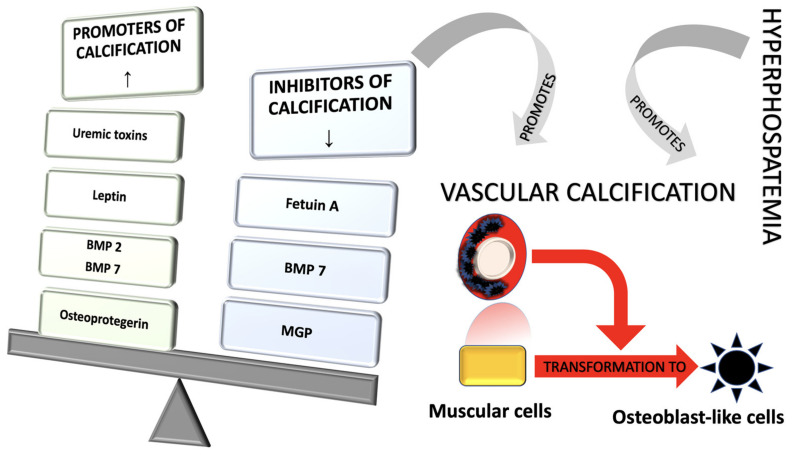
Pathophysiological mechanisms of vascular calcification—the role of inhibitors and promoters in CKD. Legend: BMP—Bone morphogenetic proteins; MGP—Gla matrix protein.

Inhibitors of the vascular calcification process, such as fetuin A, pyrophosphate, osteoprotegerin and Gla matrix protein (MGP), have a distinct activity in patients with CKD, as illustrated in Figure 3 [77].

Fetuin A is synthesized in liver cells, and decreased levels of this compound have been shown in patients with impaired renal function, correlating with vascular calcification and CV mortality [80]. MGP shows increased activity next to atheroma plaques. Serum MGP level expresses a negative correlation with the extension of coronary artery calcification process [81]. Osteoprotegerin regulates the osteoclast activity, interfering with the nuclear factor-kB binding to the specific receptor [82]. It also expresses elevated values in subjects with CKD and is a predictor of vascular calcification. In combination with CRP, the mentioned compound can be used to estimate the risk of death in patients requiring hemodialysis [83].

Another inhibitor of vascular calcification, pyrophosphate, prevents the formation of hydroxyapatite crystals. Studies in rodents have shown that this inhibitor is synthesized by a specific enzyme, and the lack of phosphodiesterase activity has been associated with the phenotypic alteration of vascular smooth muscle cells [84].

Bone morphogenetic proteins (BMPs) are essential substances in the process of transforming smooth muscle cells at the blood vessels level. BMP-2 and bone morphogenetic protein 4 (BMP-4) stimulate osteogenic differentiation through specific transcription factors. Bone morphogenetic protein-7 (BMP-7) inhibits vascular calcification by stimulating α-type actin synthesis required for smooth muscle cells. The synthesis of BMP-7 factor is performed at the renal level, and the decline of renal function is associated with the decrease of the compound [85].

Studies demonstrate the superiority of the sevelamer phosphate chelator in ESRD compared to similar calcium-containing products. The group that benefited from sevelamer showed the absence or minimal progression of the vascular calcification process, with a better prognosis of these patients [86]. Main data regarding the uremic risk factors in CVD pathogenesis are summarized in Table 1.

## 11. Particularities of the Atherosclerotic Process in Patients with End-Stage Renal Disease

CV diseases are predictors of mortality in subjects with ESRD. It has been shown that 45–50% of deaths among patients with ESRD are caused by CV disorders and 20% of these causes were represented by CAD [104,105].

A study that monitored the characteristics of 1846 patients receiving hemodialysis observed an incidence of CAD of 40%, and during the follow-up period 43% of CV hospitalizations had a main hospitalization diagnosis unstable angina pectoris and AMI [106].

**Figure 4:**
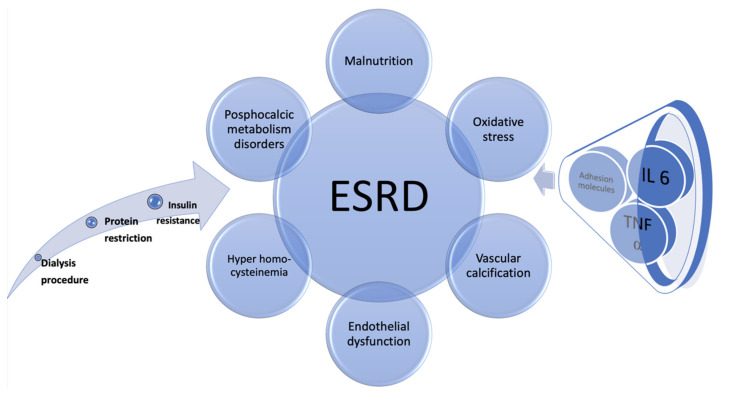
Pathophysiology of the accelerated atherosclerosis process in patients with ESRD.

Excepting the role of traditional and uremia specific CVRF, mentioned above, it was assumed that the inflammatory syndrome is more accentuated in patients with ESRD, as illustrated in Figure 4. In addition to the already mentioned biomarkers, hyper homo cysteinemia has a 90% incidence in patients with renal replacement therapy [107]. There was modest evidence of the benefits of folates and vitamin B6 and B12 complex in patients on hemodialysis, but the result of this research was not statistically significant, because of the small group of only 50 patients [108].

Malnutrition is another RF detected in patients with ESRD, with a prevalence between 18 and 75% [109]. The presence of this factor is conditioned by the hypoproteic regimen recommended for ESRD and other elements such as inflammatory syndrome, the correctness of the dialysis procedure and insulin resistance. Malnutrition has been shown to be a major predictor of mortality, especially in ESRD. The association of this RF with inflammatory syndrome and oxidative stress will contribute, along with other established RFs, to the process of accelerated atherosclerosis observed in ESRD. The common element of that association is IL-6. Thus, it has been shown that the decline in renal function is associated with increased levels of IL-6. There are studies showing that the dialysis procedure itself is responsible for the increased synthesis of IL-6, directly influencing the production of cytokines by mononuclear cells. Another important component is the dialysis membrane, which also stimulates the IL-6 synthesis. This cytokine has been shown to be responsible for initiating the atherosclerosis process in rodents with diminished activity of apolipoprotein E. The association between adhesion molecules, TNF-α and IL-6 is common in ESRD, promoting atherosclerosis [110].

CAD often manifests by silent ischemia in patients with renal replacement therapy. Coronary stenoses of over 50% were highlighted in 16 asymptomatic patients, belonging to a group of 30 patients with ESRD before the initiation of the hemodialysis procedure [94]. Similarly, another analysis that included 67 subjects with asymptomatic ESRD demonstrated hemodynamically significant coronary stenosis, involving more than 50% of the vessel lumen, in a proportion of 42%, more than half of which were in the proximal third of the epicardial coronary arteries. The CV mortality of patients with ESRD reached significant values if the systolic blood pressure (SBP) was lower than 110 mmHg. Decreased serum cholesterol, regardless of the therapeutic class used to achieve this goal, has been associated with an increased risk of death. A possible explanation is that patients with ESRD prone to MACE frequently have malnutrition, with an obvious association of classic and specific uremic CVRF. The risk of hypotension is also considerable [111].

Accelerated vascular calcification, persistent inflammatory syndrome, oxidative stress, endothelial dysfunction, malnutrition, altered phosphocalcic metabolism, are some of the mechanisms leading to an unfavorable prognosis in patients with ESRD [46]. Thus, their careful follow-up and periodic evaluation are required, especially since most of them are asymptomatic. The early identification of CAD can be the key element for improving the prognosis.

## 12. Treatment Options of ACS in CKD Patients

Pain relief is extremely important, as it leads to sympathetic activation, that determines vasoconstriction and enhances the heart workload. The most commonly used analgesics in ACS are titrated intravenous (i.v.) opioids (e.g., morphine) [112]. Methadone, fentanyl, buprenorphine, and hydromorphone are considered the least harmful opioids for the treatment of patients with advanced CKD.

In hypoxic patients with peripheral arterial saturation of oxygen (SaO_2_) <90%, oxygen therapy is recommended. Some data indicate that hypoxia may be dangerous for subjects with uncomplicated MI, because of the worsened myocardial lesion [113].

A parenteral anticoagulant, aspirin and a P2Y12 inhibitor (dual antiplatelet therapy—DAPT) should be administered to patients who are referred to primary PCI [114]. To provide total suppression of thromboxane A2-dependent platelet aggregation, aspirin can be administered orally, also by chewing, or i.v. For non-enteric-coated preparations of plain aspirin, the oral dose is required to be of 150–300 mg. Regarding optimal i.v. dosage, there are little clinical data. Considering aspirin oral bioavailability of 50%, an equivalent dose is 75–150 mg. The suppression of cyclooxygenase-2-dependent prostacyclin can be prevented with this lower dose, as indicated by pharmacological data. A recent investigation indicated that an individual dose of 250 or 500 mg acetylsalicylic acid i.v. in comparison with 300 mg orally was correlated with a rapid and more comprehensive suppression of platelet aggregation at 5 min and thromboxane generation, with similar rates of bleeding [115]. There is insufficient information on the time when P2Y12 inhibitor should be inducted in ACS subjects. Table 2 shows the results of published studies regarding the efficiency of these drugs in patients with both ACS and CKD.

β-Blockers are usually recommended in all ACS patients, unless contraindicated. In patients with AMI, atenolol, metoprolol and propranolol were evaluated, and in the context of AMI with left ventricular dysfunction carvedilol was assessed [135]. In patients with renal dysfunction, atenolol requires dose adjustment because it is renally excreted: when CrCl < 35 mL/min, dose reduction is indicated (25 mg once daily when CrCl <15 mL/min and 50 mg once daily when CrCl is 15–35 mL/min). For carvedilol, metoprolol and propranolol, no dose adjustment is needed in case of renal dysfunction, less than 5% of the oral dose being eliminated in the urine is unaltered, as they are metabolized mainly in liver [136].

Studies offer insufficient data on the use of β-blockers in ACS subjects suffering from CKD. Treatment with carvedilol was evaluated in a post hoc analysis of subjects with chronic heart failure and MI with left ventricular dysfunction. A decrease in cardiovascular and all-cause mortality was linked with carvedilol therapy in the group of patients with CKD [137].

Current guidelines advise that in ACS treatment with angiotensin-converting enzyme (ACE) inhibitors should be started and maintained in patients with diabetes, hypertension, CKD and left ventricular ejection fraction <40%, excepting cases when it is contraindicated [112,138]. The utilization of these agents is not prohibited by a specific level of serum creatinine, though when serum creatinine is greater than 2.5 mg/dL, attention is required. If serum creatinine is below 2.5 mg/dL and potassium <5.5 mEq/L, treatment with ACE inhibitors or ARBs can be taken into consideration for CKD patients [139]. Treatment with these drugs may pose problems for subjects with end-stage renal disease. In chronic dialysis patients, ACE inhibitors and ARBs have been linked to higher risk of hyperkaliemia [140].

For patients with ACS, the preferred reperfusion strategy is primary percutaneous coronary intervention (PCI) [112,138]. In reducing reinfarction, stroke or mortality, fibrinolysis is inferior to primary PCI, as highlighted by a review [141].

The technique of choice throughout primary PCI is coronary stenting. Compared to bare metal stents, drug-eluting stents (DES) lower the risk of repeated target vessel revascularization in primary PCI. Specifically, regarding lower risks of stent thrombosis and recurrent AMI, the new-generation DES have demonstrated improved safety and maintained or even enhanced efficacy compared to first-generation DES [112]. For subjects having a patent infarct-related artery, but presenting inadequate anatomy for PCI, with cardiogenic shock or a large myocardial region at risk, emergent coronary artery bypass graft surgery (CABG) should be taken into consideration. Emergent CABG is rarely carried out in STEMI patients with unsuccessful PCI or coronary occlusion not amendable to PCI, since the advantages of surgical revascularization in this context are unpredictable [112].

In acute coronary syndrome without ST segment elevation (NSTE-ACS) subjects, the main technical considerations of PCI are not different from the harmful evaluation and revascularization strategies for other CAD expressions. Implantation of new-generation DES is the standard procedure for patients with NSTE-ACS who are considered eligible for PCI in one or several vessels [138]. Table 3 illustrates the different results regarding the best choice between PCI and CABG in CKD subjects.

Current guidelines regarding STE-ACS recommendations in CKD are limited. The adjustment dose of anticoagulants and contrast agent received during PCI should be a priority. Prevention of contrast-induced nephropathy is another idea mentioned in the guidelines and measures like proper hydration, low-osmolar contrast agent usage should be considered. Fondapariux, bivalirudin, tirofiban and enoxaparin are not indicated in ESRD [112].

Among NSTE-ACS the recommendations advise precaution in anticoagulants usage and dose adaptation according to the CKD status. Hydration and low- or iso-osmolar contrast substance are mentioned as well in the NSTE-ACS guidelines. The preferred revascularization method in subjects with an acceptable surgical risk is CABG [136].

About 5–10% of NSTE-ACS patients need CABG, as they present increased risk compared to patients subjected to elective CABG [138].

More conservative treatment of cardiovascular disorders is preferred in higher risk patients (including the elderly) although the associated advantages of the therapy tend to be enhanced. Particularly for subjects with CKD, coronary angiography and subsequent revascularization could be impacted by the recognized risk of contrast substance-associated nephrotoxicity. Even though the literature indicates that a comparable percentage of subjects were considered suitable for angiography, a study has found that less patients with CKD were referred to angiography (25.2 vs. 46.8%) [147]. In clinical practice, the lack of referral to angiography because of the fear of contrast substance-associated nephrotoxicity (“renalism”) is inadequate [147].

A sub-evaluation comprising subjects from The Swedish Web-system for Enhancement and Development of Evidence-based care in Heart disease Evaluated According to Recommended Therapies (SWEDEHEART) indicated that the probability of getting reperfusion treatment for STEMI was comparable in subjects presenting normal-to-moderate renal dysfunction, and reduced in renal failure, or severe renal dysfunction. Reperfusion treatment varied from primary PCI in 71% of subjects presenting normal renal function to fibrinolysis in 58% of those presenting renal failure. According to this sub-analysis, the presence of renal failure decreases the probability of receiving the proper treatment recommended by guidelines [148].

For patients with ACS, CABG and PCI are considered alternative revascularization techniques. Because of higher thrombotic and bleeding risk, increased underlying comorbidities and coronary lesion complexity and calcification, the treatment of patients with ACS and renal dysfunction is demanding. Several meta-analyses have evaluated revascularization techniques in patients with renal dysfunction, the results being inconsistent, because the majority of the trials were retrospective, with an increased degree of heterogeneity. There is a deficiency of randomized controlled trials that evaluated revascularization techniques for patients with ACS and renal dysfunction [142].

## 13. Recent Information on Patients with ACS and CKD

An acknowledged complication of PCI, acute kidney injury (AKI), presents increased fluctuation in declared incidence because of various AKI definitions [149].

Post-contrast AKI (PC-AKI) is characterized by a decline in renal function in the days following an injection with i.v. contrast agent. The PCI-AKI usually occurs over the 48 h following the administration of a contrast agent, and normalization of renal function appears in most cases within the next 5 days. Other factors that may lead to AKI, like embolic disease, hypotension and medication, should be taken into account [150]. Table 4 presents the criteria that define PCI-AKI and risk factors for it.

PCI is the gold standard treatment of patients with ACS. A retrospective study highlighted that in 99 subjects, PCI-AKI appeared in each 20th patient with STEMI, although in the group of subjects with CKD nearly 10% of patients were affected. Significantly increased mortality rates were seen in patients with PCI-AKI [159]. A careful assessment of the risk factors for PCI-AKI should be performed in patients who will undergo contrast-enhanced diagnostic or interventional procedures.

PCI-AKI can lead to adverse effects, as shown by a retrospective study. AKI may complicate with chronic renal malfunction and higher risk of long-term adverse effects [160].

Up to date there are no adequate studies to indicate if preventing PCI-AKI ameliorates mortality, yet it decreases the prevalence of CKD. Decreasing the occurrence of AKI may enhance long-term results by lowering CKD and mortality [150]. The use of low osmolarity or iso-osmolar contrast, decreasing the volume of contrast substance, and the use of non-iodine contrast media are adjustable factors. The risk of PCI-AKI is correlated with the quantity of contrast agent administered [150]. Both the direct renal tubular toxic action and the indirect intra-renal hemodynamic modifications may be prevented by intravascular volume expansion when administering the contrast agent. This is likely to reduce tubular fluid viscosity and may decrease cellular injury by diluting the tubular level of contrast agent. Even more important, it may adjust and improve other related causes of AKI, unconnected to contrast agent, for instance dehydration [150].

For preventing PCI-AKI, N-acetylcysteine, either oral or i.v, was thoroughly studied, without consistent benefits [161].

Limited data to sustain the interruption of treatment with diuretics, ARBs or ACE inhibitors, is available. For patients with GFR <40 mL/min/1.73 m^2^ and CKD, National Institute for Health and Clinical Excellence guideline recommends taking into account the temporarily cessation of these medications [162].

After contrast agent administration, it is recommended to avoid or cease nephrotoxic medications [163], including NSAIDs. Treatment with metformin must be temporarily interrupted after the contrast agent is administered, because of the risk of lactic acidosis [150]. In patients at high risk of AKI, prophylactic renal replacement therapy is not advisable for the elimination of contrast agent. The excretion of contrast agents is delayed in patients with renal dysfunction [150]. By intermittent hemodialysis, these contrast agents can be efficiently removed in a proportion of about 60–90%. A decrease in the prevalence of post-contrast AKI with prophylactic renal replacement therapy is not indicated by available data, and it is not recommended by Kidney Disease Improving Global Outcomes (KDIGO) [150].

Trans radial access is preferred over transfemoral access to minimize bleeding risk, according to data from recent randomized controlled studies. However, in patients with advanced CKD, who may be candidates for arteriovenous fistula for dialysis, the trans radial approach may be less preferred, because of the risk of radial artery occlusion or stenosis [164].

After cardiac surgery, a critical usual complication is represented by cardiac surgery-associated acute kidney injury (CSA-AKI). CSA-AKI is among the most powerful predictors of mortality and can delay the recovery in about 30% of patients [165]. In a retrospective study, patients referred to cardiac surgery did not have a worse prognosis or an increased risk of CSA-AKI in the next 7 days [165]. The ideal moment of coronary angiogram prior to surgery must be assessed taking into consideration the severity of the cardiovascular illness and risk factors for CSA-AKI in subjects predisposed to AKI. In order to elaborate guidelines, well-designed prospective trials should be undertaken with greater number of cases. Also, a functional assessment model according to the clinical indices and new renal biomarkers should be determined [165].

There is insufficient data to indicate the administration period for antiplatelet therapy in patients with CKD subjected to PCI. Due to the risk of ischemic and bleeding complications in patients with CKD, this is a major concern and justifies further studies. In patients with advanced CKD, a prolonged duration of antiplatelet therapy may be linked to severe bleeding risk and uncertain advantages. For ESRD and/or CKD patients with atrial fibrillation subjected to PCI, no information from randomized controlled or prospective studies about the management of anticoagulant or antiplatelet treatment is available [166].

Higher potency P2Y12 inhibitors were administered in about one third of CKD patients in the post-AMI population [167]. A higher risk of moderate or severe bleeding following discharge was seen in patients with advanced CKD [167]. Increased rates of early discontinuation were registered in patients with CKD discharged on an increased potency P2Y12 inhibitor compared to those discharged on clopidogrel [167]. Patients with an advanced degree of CKD were not more frequently predisposed to a premature interruption of P2Y12 inhibitor treatment, although they were more probable to temporarily discontinue the treatment with P2Y12 inhibitor because serious bruising or bleeding and to change the treatment than patients with less serious or no CKD [167].

In patients with CKD in whom the risk of bleeding could be significant, a meta-analysis highlighted the limited effectiveness in preventing ischemic events noted with the use of extended DAPT. Compared to longer DAPT, shorter DAPT is not apparently inferior in patients with DES and CKD [166].

A recent investigation in subjects with CKD evaluated the clinical effectiveness and safety of various antiplatelet treatment schemes with increased platelet inhibition activity compared to a regular dose of clopidogrel-based DAPT. The clinical results were significantly improved by antiplatelet treatment schemes with enhanced antiplatelet action over standard clopidogrel-based DAPT, including MACE (RR 0.79, *p* < 0.00001), all-cause mortality (RR 0.67, *p* = 0.003), MI (RR 0.28, *p* = 0.0007), without significant bleeding (RR 1.14, *p* = 0.33) in patients with CKD and ACS. In patients with severe CKD (eGFR <30 mL/min) the risk of bleeding was significantly higher than in moderate CKD. Antiplatelet treatment with ticagrelor, prasugrel or triple therapy can considerably suppress platelet activity in patients with CKD, even in those on hemodialysis, but double dose clopidogrel does not [168].

Some patients with CKD stage 5 can benefit from shorter DAPT and others may require extended treatment, considering the risk of bleeding versus thrombosis in ESRD. A prudent approach is advisable for each patient with ESRD, as evidence for extended DAPT or arguments to reduce DAPT may emerge. The acute in contrast with the elective presentation also appears to be significant when choosing the antiplatelet treatment, as noted by the authors. In the future, decision pathways established by artificial intelligence algorithms may provide personalized DAPT regimens for each patient, in a way not currently possible when using guideline directions [169].

## 14. Conclusions

The increased mortality and negative prognosis of patients with CKD correlate with the existence of an accelerated atherosclerosis process, involving complex pathophysiological mechanisms, initiated by classical CVRF and RF specific to the uremic environment. The identification of patients with impaired renal function likely to develop CV complications involves their regular monitoring and evaluation, for an early identification of both categories of RF, and the initiation of appropriate treatment. The treatment of anemia, regression of proteinuria by using certain therapeutic classes, or use of sevelamer to influence the process of vascular calcification may be therapeutic solutions that could influence the evolution of CAD in patients with CKD.

The lack of clinical evidence, because of the exclusion of patients with CKD and ACS from major studies, makes it difficult to establish the best treatment options and the most appropriate methods of myocardial revascularization for these patients. New studies addressing these objectives and the particularities of CAD in the context of impaired renal function are needed.

Renal nihilism is characterized by frequent utilization of conservative treatment in ACS or improper use of lower drug doses, because of impaired renal function. Limiting this phenomenon and knowing the complex pathophysiological interaction between classic CVRF and RF specific to the uremic environment represent the necessary premises for increasing the treatment quality and prognosis in patients with CKD and CAD.

## 15. Future Perspectives

Discovering the pathophysiological mechanisms existing between CKD and CAD represents an important step towards finding specific treatment options. Even though the most common cause of death in CKD patients is represented by CV disease, the therapeutic options are identical with those from general population, without targeting the uremic RF or a certain relationship responsible for the mentioned association.

ESRD is a special category and clear data regarding their treatment in the context of CAD is needed. A major predictor of the ESRD outcome is represented by vascular calcification. Trials demonstrating the certain effect on the CV events and improving the ESRD prognosis once this uremic RF is properly controlled or eliminated are lacking. The available limited data consists of retrospective observational studies that illustrates a favorable effect of non-calcium-containing phosphate binder [170,171].

Prevention of CV disorders in CKD subjects is another important element and all the efforts should pursuit an accurate management of the uremic RF and adequate paraclinical methods that detect early their presence. Intrastent thrombosis in CAD patients that associate CKD represents a reality. Beside the type of stent used, a proper strategy of controlling uremic RF may contribute to preventing the complication [170].

Platelet reactivity is variable in CKD subjects and a recent published study illustrated that extended therapy with potent P2Y12 receptor inhibitors did not have major influence regarding the primary endpoints [172]. Future observations analyzing the indication of prolonged antiplatelet therapy and outcome in CKD are required.

Antiplatelets effects and their real action in CKD subjects represents a potential issue for an ongoing study. Specific measurements of platelets reactivity are also involved and the correlation of their values with CV disease prevention is an objective. The weak points or the strengths of the current antiplatelet therapy in CKD is going to be revealed due to this study. Exclusion criteria involves subjects with recent ACS, revealing the fact that the study is focused on primary prevention of CV disorders using antiplatelet drugs [173].

Ongoing analysis regarding usage of ticagrelor or clopidogrel in severe and terminal CKD subjects with interventional myocardial revascularization for ACS will solve the dilemma regarding best treatment choice [174].

Another interesting perspective is proposed by a study that explored the properties of the vascular dose of rivaroxaban in preventing CV disease [175]. The expected results will probably have a major impact in CV disease prevention, especially in CKD.

The concepts of patient-reported outcome (PROMs) and patient-report experience measure (PREMs) are suitable for improving quality of life in CKD subjects. Creative solutions like using questionnaires known as PROMs and finding the real symptoms of patients may consist of an improvement in CKD standard of care. Also, PREMs may identify the problems that require improvement for a better hospital performance. Evidence regarding PROMs revealed that a shorter duration of dialysis procedures repeated more often was associated with a better health-related quality of life compared with standard hemodialysis protocols [176]. Implementation of PROMs and PREMs may be challenging but their contribution to improving the relationship between patients and medical system is impressive [170]. Patient’s opinion along with the opportunity to collaborate with the physicians and participate in choosing the best medical option can improve the prognostic of their disease and increase treatment adhesion.

## Figures and Tables

**Table 1:** Main studies revealing the importance of uremic risk factors in cardiovascular disease pathogenesis.

Number of Patients	Mechanisms Incriminated in Cardiovascular Disease Development	Study Outcome	Findings	Ref.
**Anemia**				
3015	Increased preload Reduced afterload Increased cardiac output	MI; Fatal CHD; Stroke; Death	Interaction between anemia and CKD was statistically significant for all outcomes.	[84]
14,410		CV disease	Anemia is associated with CV disease and represents an independent predictor for their outcome.	[85]
67,000		CV mortality	Decreased levels of hematocrit were associated with high CV mortality	[86]
**Inflammation**				
244	Decline of the cytokines’ clearance Inflammatory cells recruitment Vascular calcification by transforming endothelial cells into osteoblasts-like cells Acceleration of atherosclerosis	Coronary events	Inflammatory markers predict coronary events in women with CKD	[87]
585		CV death	Inflammation is an independent predictor of CV mortality in CKD	[88]
225		CV events; Death	High ADMA levels are correlated with CV events and death in ESRD	[89]
3905		Development of vascular events: MI, stroke	High levels of hs-CRP predict vascular events in CKD patients	[90]
2399		Prediction of CV disease occurrence; Death	Inflammation is an independent predictor of CV disease development and death in CKD	[91]
10,000		Major CV events; CV death; All-cause mortality	Improved outcome in CKD patients using an inhibitor of interleukin-1β	[92]
**Proteinuria and Microalbuminuria**				
870	Association with increased production of adhesion molecules Promotes endothelial dysfunction	CAD	Micro and macroalbuminuria represents independent predictors for CAD development	[93]
920,985		All-cause mortality; MI; Progression of kidney function decline	High values of proteinuria are independently associated with the mentioned outcomes	[94]
25,112		Fatal and stable CAD	Microalbuminuria is useful in detecting subjects with high risk of CAD development	[95]
9043		CV events (MI, stroke, CV death)	All the degrees of albuminuria predict CV events occurrence in subjects with or without DM	[46]
**Oxidative Stress and Endothelial Disfunction**				
173	Reactive oxygen species reduces the available nitric oxide There is an enhancement of phagocytic cells infiltration in the vascular wall	Correlation between oxidative stress markers and CV disorders in subject with and without CKD	Markers of oxidative stress influence CV disease appearance in CKD	[96]
3375		Correlation between myeloperoxidase and CAD development	The mentioned oxidative stress marker predicts CAD appearance in health subjects	[97]
**Vascular Calcification**				
202	Vascular calcification expresses similar characteristic with osteogenesis Disbalance between promoters and inhibitors of vascular calcification, favorizing the promoters Increased arterial stiffness Impaired coronary artery perfusion	CV and all-cause mortality	Arterial calcification represents an independent predictor of CV mortality	[98]
2069		CV disease development All-cause mortality	Coronary artery calcification is strongly related with CV disorders, especially MI and all-cause mortality in CKD	[99]
117		Coronary artery calcification burden in CAD using autopsy cases	Different types of coronary artery calcification are correlated with the uremic RF, especially medial calcification	[100]
17,222		CV mortality CV events	Coronary artery calcification is an independent predictor of CV events and mortality in CKD patients	[101]
**Malnutrition**				
291	Association with an important inflammatory status The most important consequences are hypoalbuminemia and dyslipidemia	CV disease risk	Strong correlation between malnutrition and CV disease occurrence	[102]
100		Atherosclerosis and mortality	Inflammation-malnutrition score is useful for atherosclerosis and negative outcome prediction	[103]

Legend: CKD—Chronic kidney disease, CHD—Coronary Heart Disease, CV—Cardiovascular, ESRD—End-stage renal disease, MI—Myocardial infraction.

**Table 2:** Relevant findings regarding treatment options in ACS and associated CKD.

Design/No. of Subjects	CKD Stage	Type of ACS	Treatment Group/Control Group	Efficacy	Ref.
**Antiplatelets**					
Prospective *, **/2070	CKD stages 1 to 5	With or without CAD	Low-dose aspirin users/Aspirin non-users	Beneficial effect in reducing CV events /mortality; no significant increasing of bleeding risk in subjects with previous cardiac disorders; recommended for secondary prevention.	[104]
Sub analysis **/28,320	ESRD	With or without CAD	Aspirin users/Aspirin non-users	No improvements in prevention of CV mortality/cardiac events, potential benefits in strokes prevention; no increase of bleeding risk.	[105]
Sub analysis **/3237	CKD stages 1 to 5	STEMINSTEMI	Ticagrelor/Clopidogrel	In patients with both ACS and CKD, ticagrelor (vs. clopidogrel) significantly decreases ischemic endpoints, respectively mortality, without a major bleeding risk, but involving much more minor bleedings events.	[106]
Sub analysis **/1484	Moderate GFR	AMI	Statins + aspirin/Aspirin alone or no treatment	Chronic treatment with statin or the combination of aspirin + statin was associated with reduced in-hospital mortality and better results, both in the short and long term.	[107]
Sub analysis **/2490	CKD stages 1 to 5	All typed of ACS	Potent P2Y12 inhibitor (Prasugrel or ticagrelor) /Clopidogrel	Ticagrelor vs. clopidogrel or prasugrel decreases the recurrence of MI as well as mortality (occurring from any cause) in subjects with ACS + CKD undergoing PCI. The therapy has been shown to be safe, ensuring the long-term maintenance of the low risk of bleeding.	[108]
Meta-analysis **/31,234	CKD stages 1 to 5	All typed of ACS	Potent P2Y12 inhibitor (Prasugrel or ticagrelor) /Clopidogrel	In patients suffering of both ACS + CKD, PPAs (vs. clopidogrel) are considered to have a significantly lower incidence of MACE, without increasing the risk of bleeding events. In contrast, in PCI subjects, the beneficial effect of PPAs on MACE has been associated with decreased mortality.	[109]
Prospective study **/60	≥3 b CKD	NSTE-ACS	Ticagrelor/Clopidogrel	In patients with CKD and NSTE—ACS, ticagrelor proved an increased potent platelet inhibition vs. clopidogrel	[110]
Sub analysis **/2171	CKD stages 1 to 5	ACS and stable CAD	1-month DAPTTicagrelor monotherapy (23 months)/12 months DAPT12 months aspirin	In patients with CKD treated with ticagrelor monotherapy, no differential therapeutical side effects were found related to all-cause death/new Q-wave AMI after PCI	[116]
Retrospective study/7718	ESRD	ACS and stable CAD	Prasugrel or ticagrelor/Clopidogrel	In patients with ESRD managed with drug-eluting stents, it was observed that ticagrelor or prasugrel (vs. clopidogrel) cannot be associated with relevant benefits	[111]
**Statins**					
Sub analysis**/4491	Moderate CKD	With or without CAD	Pravastatin/Placebo	In patients with/at risk for coronary disease + moderate CKD, pravastatin decreases CV event rates	[117]
Prospective study *, **/1255	ESRD	With or without CAD	Atorvastatin/Placebo	No significant difference in the CV events rate/total mortality in the therapy group	[118]
Meta-analysis *, **/3594	CKD 1 to 5	With or without CAD	Statins/Placebo	In patients with non-dialysis CKD, statin treatment significantly alters the lipid profile; also, a less beneficial effect was observed in dialysis patients, the long-term therapy being less effective.	[119]
Retrospective study **/510	CKD 1 to 4	ACS	Statins/Placebo	In patients with ACS + CKD, statin therapy significantly reduced CV events.	[120]
Sub analysis **/8945	CKD 1 to 4	ACS	Statins/Placebo	In patients with ACS + CKD, it was observed that the beneficial effect of statins was maintained.	[121]
**β-Blockers**					
Prospective study **/1724	CKD 1 to 4	ACS	β-Blockers/Placebo	Decreased CV mortality in subjects receiving both β-Blockers.	[122]
Prospective study **/3510	CKD 1 to 4	ACS	β-Blockers/Placebo	Improving of 1 year survival rate was noticed in subjects receiving β-Blockers regardless of the kidney function status.	[123]
Sub analysis **/3075	CKD 1 to 4	ACS and stable CAD	β-Blockers/Placebo	AMI rate and sudden cardiac death were reduced due to β-Blockers regardless of the kidney function.	[124]
Retrospective study **/146765	ESRD	ACS	β-Blockers/Placebo	In subjects with ESRD + ACS, β-Blockers reduced the mortality with 22%.	[125]
**ACE inhibitors/ARB**					
Sub analysis **/64,442	CKD 1 to 5	ACS	ACE inhibitors or ARB/Placebo	Reduced mortality and improved outcome in subjects treated with ACE inhibitors or ARB.	[126]
Meta-analysis *, **/56,694	CKD 1 to 4	With or without CAD	ACE inhibitors or ARB/Placebo	ACE inhibitors had a major contribution in improving CV mortality and CV events, but ARB did not influence those outcomes.	[127]
Retrospective study **/527	ESRD	With or without CAD	ACE inhibitors/Placebo	Reduced mortality in ESRD subjects treated with ACE inhibitors.	[128]
Meta-analysis **/81,541	CKD 1 to 4	With or without CAD	ACE inhibitors/Placebo	Better survival rate and reduced mortality in subjects using ACE inhibitors.	[129]
Meta-analysis **/33,960	CKD 1 to 4	CAD	ACE inhibitors/Placebo	ACE inhibitors reduced the mortality and CV events rate in subjects with stable CAD without LV dysfunction.	[130]
**Anticoagulants**					
Prospective study **/1724	CKD 1 to 4	STEMI	UFH/Enoxaparine	Enoxaparine was superior to UFH, but in subjects with severe CKD this effect disappeared, and adverse effects rate was high.	[131]
Sub analysis **/1915	CKD 1 to 4	NSTEMI	Tirofiban + UFH/Tirofiban Placebo + UFH/UFH Placebo + Tirofiban	Reduced complications of ACS in subjects treated with mild or moderate CKD treated with Tirofiban.	[132]
Meta-analysis **/5035	CKD 1 to 5	CAD	Bivalirudin/Heparin	Benefits in reducing ischemic and bleeding events in subjects using Bivalirudin.	[133]
Sub analysis **/12,939	CKD 1 to 4	ACS	Bivalirudin/Heparin + glycoprotein IIb/IIIa inhibitor/Bivalirudin + glycoprotein IIb/IIIa inhibitor	Monotherapy with bivalirudin therapy did not influence the ischemic outcomes, but the bleeding events were lower.	[134]

Legend: ACE inhibitors—Angiotensin-converting enzyme inhibitors, ARB—angiotensin receptor blockers, CV—Cardiovascular, LV—Left ventricle, MACE—Major adverse cardiovascular events, PPAs—P2Y12 receptor antagonists, UFH—Unfractionated heparin, *—primary prevention, **—secondary prevention.

**Table 3:** Comparison of interventional and surgical revascularization in patients with CAD and CKD.

Design/Origin of the Study Population/No. of Subjects/Type of Stent Used/Type of CAD	Patients with		Short/Long Term MACE			Findings	Ref.
	**Left Main Disease**	**3 Vessels Involvement**					
	PCI; CABG		PCI%	CABG%	*p* Value		
Retrospective cohort study/The Second Drug-Eluting Stent Impact on Revascularization Registry/2923/Second generation DES/ACS	79 (4.2%); 185 (18.1%)	501 (26.3%); 707 (69.3%)	1.5/12.2	4.4/10.0	<0.0001/ 0.1124	The revascularization technique was recognized to be a time sensitive covariate for MACE. In the short term, when compared to CABG, PCI is likely to present a decreased risk for MACE. Risk ratio for PCI compared to CABG grows with time.	[142]
Observational study/Cardiac Care Network of Ontario Cardiac Registry/1786/Second generation DES/Stable CAD and ACS	132 (13.3%); 1108 (36.8%)	292 (29.3%); 1679 (55.8%)	13.8/40.3	4.0/18,6	<0.001/ <0.001	CABG was linked with enhanced early and late clinical results in contrast with PCI using DES in subjects who present CKD subjected to index revascularization.	[143]
Observational study/New York State Percutaneous Coronary Intervention Reporting System and the Cardiac Surgery Reporting System registries/5920/Second generation DES/Stable CAD and ACS	1269 (42.9%); 1367 (46.2%)	-	29/458 death events	51/469 death events	0.01/ 0.40	PCI was linked to an increased long-term risk of recurrent revascularization and maybe AMI, while CABG was linked with an increased short-term risk of stroke, repeated revascularization or death.	[144]
			11/90 stroke events	50/143 stroke events	<0.0001/ 0.0002		
			20/233 AMI events	15/153 AMI events	0.40/ <0.0001		
Retrospective sub-analysis/Syntax/1638/First generation DES/Stable CAD and ACS	19 (18.5); 13 (11.7)	31 (34.4%); 20 (24.5%)	6.3/37.3	4.0/25.1	0.34/ <0.001	After PCI, the negative influence of CKD on long-term results seems to be enhanced in contrast with CABG, particularly in CKD subjects with extensive CAD and diabetes.	[145]
Retrospective sub-analysis/Excel/361/Second generation DES/Stable CAD and ACS	177; 184	64	19/40	54/33	<0.0001/ <0.001	No differences between the PCI or CABG for the components of death or primary composite endpoint, stroke, or AMI at 3 years even though PCI was linked with considerably decreased short-term MACE in contrast to CABG. PCI and CABG are both indicated in elected high-risk patients with CKD and LMCAD.	[146]

Legend: CAD—Coronary artery disease, DES—Drug-eluting stent, MACE—Major adverse cardiovascular events, LMCAD—Left main coronary artery disease.

**Table 4:** Definition and risk factors of post contrast AKI.

		Criteria	Ref.
**Definition**	The condition can be linked causally to giving contrast media	Serum creatinine increases by ≥26.5 μmol/L within 48 h; Serum creatinine increases by ≥1.5-fold from baseline within 1 week.Urine output <0.5 mL/kg/h of body weight for >6 consecutive hours.	[151]
**Risk Factors**	Individual factors	Age	[152]
		Diabetes mellitus	
		Myocardial infarction	
		Chronic kidney disease	
		Cardiogenic shock	
		Hyponatremia	
		Anemia	[153]
		Leukocytosis	[154]
		Previous treatment with renin-angiotensin-blockers	[155]
	Characteristics of the contrast agent	Contrast dose/100 mL	[156,157]
		High-osmolality contrast	[158]
